# Factors Associated with Breast Cancer Awareness in Thai Women

**DOI:** 10.31557/APJCP.2019.20.6.1825

**Published:** 2019

**Authors:** Cameron Paul Hurst, Supannee Promthet, Nitchamon Rakkapao

**Affiliations:** 1 *QIMR Berghofer medical Research Institute, Queensland, Australia, *; 2 *Faculty of Public Health, Lampang Campus, Thammasat University, Lampang, *; 3 *Faculty of Public Health, Khon Kaen University, Khon Kaen, Thailand. *

**Keywords:** Breast cancer, breast cancer awareness, B-CAS, Thai women

## Abstract

**Background:**

Breast cancer is the most common cancer in women worldwide. In south-east Asia, both the incidence and mortality rates of breast cancer are on the rise, and the latter is likely due to the limited access to large-scale community screening program in these resource-limited countries. Breast cancer awareness is an important tool which may, through increasing breast self-examination and the seeking of clinical examination, reduce breast cancer mortality. Investigating factors associated with breast cancer awareness of women is likely to help identify those at risk, and provide insights into developing effective health promotion interventions.

**Objective:**

To investigate factors associated with breast cancer awareness in Thai women.

**Methods:**

A cross-sectional sample of Thai women aged 20-64 years was collected during August to October, 2015 from two provinces of southern Thailand (Surat Thani and Songkla). A questionnaire including the Breast Cancer Awareness Scale along with demographic characteristics was administered and Proportional Odds Logistic regression was then used to investigate factors associated with breast cancer awareness.

**Results:**

In total, 660 Thai women participated in this study. Factors most often associated with the various breast cancer awareness domains were age and rurality. While rural women had poorer knowledge of breast cancer signs and symptoms, they also had lower levels of perceived barriers and considerably better breast cancer awareness behaviors.

**Conclusion:**

Despite lower knowledge of breast cancer risk factors and no evidence of better knowledge of signs and symptoms, we found rural Thai women had considerably better breast cancer awareness behavior. This may be due to these women’s lower levels of perceived barriers to breast cancer screening services. Indeed this suggests, at least in Thai women, that interventions aimed at lowering perceived barriers rather than enhancing disease knowledge may be more successful in engaging women with breast cancer screening services and increasing breast self-examination.

## Introduction

Despite a considerably higher breast cancer incidence in western populations such as those in Australia and New Zealand, North America and Western Europe when compared with Asian populations (GLOBOCAN, 2018), early diagnosis and improvements in treatment have led to a considerable fall in breast cancer case fatality rates in these western populations. In contrast, breast cancer mortality is increasing in many developing countries and is the most frequent cause of cancer death in women (Ferlay et al., 2015; Ghoncheh et al., 2016). In south-east Asian countries, despite comparatively lower breast cancer incidence rates, the mortality rate remains high. For example, while the cumulative risk of breast cancer incidence in south-east Asian countries is about half that of North American countries (4.17% vs 9.32%), the cumulative risk of breast cancer mortality is higher (1.61% vs 1.38%; GLOBOCAN, 2018). This higher mortality rate is likely to be due to the poorer screening and treatment practices associated with resource-limited health care systems in many of these countries. Indeed, many women diagnosed with breast cancer in resource-limited Asian countries are diagnosed at an advanced stage of the disease (Norsa’adah et al., 2011; Buranaruangrote et al., 2014), and this is likely to be one of the main causes of the higher breast cancer case fatality rate. Moreover, trends of breast cancer incidence in many developing Asian countries continue to rise, potentially compounding the problem of high breast cancer mortality. In the last 10 years, countries like China and India have seen considerable increases in breast cancer incidence (Parkin et al., 2005; Ghoncheh et al., 2016). As with other resource-limited countries, diagnosis of breast cancer in Thai women is typically at a comparatively advanced stage of the disease (Buranaruangrote et al., 2014; Poum et al., 2014).

Several studies have suggested that the increasing incidence of breast cancer in Asian women is attributable to economic development and adaptation of a more westernized lifestyle, including delayed childbirth, reduced parity and breastfeeding, weight gain, and increased consumption of animal fat (Moore et al., 2010; Forouzanfar et al., 2011). However, it is also possible the impact of the various breast cancer risk factors may differ in Asian women. For example, peak breast cancer incidence in Asian women has been found to be between 40 to 50 years of age, whereas in Western countries the highest incidence is in women aged 60 to 70 years (Youlden et al., 2012). 

Earlier detection of breast cancer leads to more effective treatment, in turn, leading to higher survival rates. Publicly funded, community-based mammographic screening has been broadly accepted as one of the best methods of reducing breast cancer mortality (Harris et al., 2011). However, such programs are currently infeasible in many resource-limited countries. For instance, while approximately 78% of high-income countries provide community-based mammographic screening programs, only 48% of upper-middle income countries have such programs (Bellanger et al., 2018). Other methods for the early detection of breast cancer need to be considered in the resource-limited health setting. 

The efficacy of breast self-examination (BSE) in reducing breast cancer mortality remains controversial. For example, in some studies BSE could not be shown to reduce breast cancer mortality (Semiglazov et al., 1993; Newcombe et al., 1991). However, these studies were conducted in resource-sufficient western countries, where the impact of BSE, on top of community-based screening programs, may not have a substantial effect on reducing mortality. In countries lacking comprehensive community screening programs, BSE may have a substantial effect acting as a trigger for women seeking earlier clinical examination, thereby leading to earlier detection. In resource-limited countries, it is individual women, rather than the health-care system, that is likely to be the main agent in women seeking clinical breast examination or mammography. Several studies have shown that breast cancer awareness is associated with BSE (Thornton et al., 2008; Radi, 2013; Al-Khamis, 2018). Indeed, one previous study demonstrated that breast cancer awareness was a strong predictor of BSE with a sensitivity of 83.3% and specificity of 60.2% in Thai women (Rakkapao et al., 2017). Consequently, identifying those at risk of poor awareness is an important first step in raising breast cancer awareness, increasing breast self-examination, and subsequently, women seeking clinical breast examination in a timely manner. 

Several studies have investigated factors associated with breast cancer awareness, however, most of these studies have used instruments that have not been rigorously validated (Liu et al., 2014; Sathian et al., 2014), or instruments that were developed for western populations unlikely to be valid outside of these contexts (Linsell et al., 2010). In this study we investigate the factors associated with breast cancer awareness in Thai women. We employ a psychometrically-validated instrument, the Breast Cancer Awareness Scale (B-CAS), which has been shown to be valid in Thai women (Rakkapao et al., 2016; Rakkapao et al., 2017). 

## Materials and Methods

*Study design and sample*

In this cross-sectional study, 660 Thai women aged 20-64 years were sampled from August to October, 2015. Stratified random sampling was used to select participants from rural and urban areas of the Surat Thani and Songkla provinces of southern Thailand. Stratification was based on locality-age groups as reported by the Department of Provincial Administration statistics (2013). Women were approached at primary health care units within their respective locality (rural/urban) and province (Surat Thani / Songkla) districts, and upon providing informed consent, recruited into the study. Those women with a personal history of breast cancer, currently pregnant or breast feeding, or not literate in the Thai language were excluded. Permission to collect the data was obtained from the head of each community, and all participants provided informed consent. The study protocol was approved by the ethics committees of Khon Kaen University (HE 582053).

*Variables measured*

Data were collected using a self-administered questionnaire which included demographic variables along with the Breast Cancer Awareness Scale (B-CAS), an instrument previously validated in this population (Rakkapao et al., 2016; Rakkapao et al., 2017). The B-CAS instrument contains five domains: knowledge of breast cancer risk factors, knowledge of breast cancer signs and symptoms, attitude to breast cancer prevention, barriers of breast screening, and health behavior related to breast cancer awareness. B-CAS content was validated using a panel of twelve experts with extensive experience working in the breast cancer field (Rakkapao et al., 2016). Construct validation was performed using confirmatory factor analysis where the measurement model was shown to fit the data well (Rakkapao et al., 2017). Criterion validity of the B-CAS subscales was evaluated in terms of the subscales’ ability to discriminate between women who do, and don’t regularly conduct breast self-examination. All five subscales demonstrated utility in identifying women who do not perform breast self-examination (Sensitivities ranging from 65.4% to 83.3%). Test-retest and Internal consistency reliability for all subscales was also shown to be strong (Rakkapao et al., 2016; Rakkapao et al., 2017). 

*Statistical analysis*

Epidata (v3.1; Lauritsen and Bruus, 2004) was used to enter the data, and the logic check mode was used to check for data errors. Demographic characteristics of participants were summarized using means and standard deviations, for continuous variables, and frequencies and percentages, for categorical data. In the absence of clinically meaningful cut-points, the overall scale and each individual B-CAS domain was split into tertiles with women in the bottom 33% scored as low, the middle tertile as medium, and the upper tertile as high. Proportional odds logistic regression was then used to investigate the factors associated with each B-CAS domain, with bivariate and multivariable models used to generate unadjusted and adjusted odds ratios, respectively. The proportionality assumption of the proportional odds logistic regression was assessed graphically from the bivariate models of each outcome-predictor combination and the results of these models (Odds ratios, 95% confidence intervals and p-values) are provided in Supplementary Tables S1 through to S6. All analysis was conducted using the R statistics package (R Core Team, 2018) and a significance level of 0.05 was used throughout all inferential analysis.

## Results

A total of 660 Thai women completed the questionnaire (Response rate: 94.3%), and their ages ranged from 20 to 64 years old (Mean=41.38, SD=11.92). Most participants were aged between 35 to 59 years old (58.84%), had not achieved more than a high school education (74.3%), and resided in rural areas (69.85%). Demographic characteristics of the participants are presented in Table 1.

The adjusted odds ratios of Breast Cancer Awareness scale (overall) and B-CAS subscales are provided in Tables 2 and 3. The unadjusted estimates generated from the bivariate analysis are provided in the Supplementary Tables S1-S6.

After adjusting for other potential associated factors, rural living environment had a substantial effect of overall B-CAS (Table 2) with rural women having considerably lower odds of better awareness relative to urban women (OR adjusted=0.43; 95%CI: 0.29,0.62; p<0.001). Another significant effect was marital status (χ^2^=6.94, df=2, p<0.05) with married women exhibiting higher odds of better breast cancer awareness, relative to single women (OR adj = 1.60; 95%CI: 1.03, 2.48; p<0.05), although those that had been previously married did not differ significantly from single women. (Table 2) 

Rural women have a substantially higher risk of poorer knowledge of breast cancer risk factors (Table 2) with the odds of better knowledge of breast cancer risk factors in rural women only 0.40 times that of urban women (OR=0.40; 95%CI; 0.27,0.59; P<0.001). In terms of occupation, the odds of better knowledge of risk factors of breast cancer in somebody with government official occupation is 0.25 times that of those working in the agricultural sector (OR=0.25, 95%CI; 0.11, 0.53; P<0.001) and the odds of better knowledge of risk factors of breast cancer in the unemployed is 0.41 times that of agricultural workers (OR=0.41, 95%CI; 0.19, 0.87; P<0.001). Although the omnibus Likelihood ratio test suggested that marital status is associated with knowledge of risk factors, the local Wald tests failed to identify which groups may differ.

After mutual adjustment of all covariates, only family history of (any) cancer could be shown to be associated with knowledge of breast cancer symptoms. (Table 2). The odds of better knowledge of breast cancer symptoms is 62% less in women with a family history of cancer, relative to those with no family history of cancer (OR=0.38; 95%CI; 0.16,0.95; P<0.05).

Few factors were associated with attitude to breast cancer prevention (Table 3). However, the odds of better attitude to breast cancer prevention in rural women is 40% less relative to that of urban women (OR=0.60; 95%CI; 0.41, 0.87; P<0.001). 

Older women were less likely to perceive barriers to breast cancer screening as a problem with the odds of higher perceived barriers of breast screening decreasing 24% for every 10 years of age (OR=0.76, 95%CI: 0.64, 0.91; P<0.05; Table 3). Similarly, rural woman tended to have lower risk of a higher perception of breast cancer screening barriers with the odds of higher perceived barriers 44% less for rural women relative to urban women (OR=0.56; 95%CI: 0.37, 0.84; P<0.05). In our sample, higher level of education also seemed associated with lower perceptions to barriers of breast cancer screening. However, we could only demonstrate that those with a high school education had significantly lower odds of higher perceptions of barriers, relative to those with only a primary school education (OR=0.44, 95%CI: 0.28, 0.68; P<0.05). 

Many factors were associated with health behavior related to breast cancer awareness (Table 3). As a woman ages 10 years the odds of better health behavior is 1.63 times higher (95%CI; 1.36, 1.95; P<0.05). The odds of better health behavior related to breast cancer awareness was 4.73 times higher in rural women relative to urban women (OR=4.73; 95%CI; 3.08, 7.27; P<0.05). In terms of education, the odds of better health behavior in those with high school is 3.8 times higher than those with a primary school or less (OR=3.8, 95%CI; 2.42, 5.96; P<0.001) and the odds of better health behavior in somebody with diploma or equal is 5.97 times higher relative to those with primary school or less (OR=5.97, 95%CI; 2.85, 11.77; P<0.001). 

**Table 1:** Demographic Characteristics of Participants

Characteristics	n=660	%
Age group		
Early adulthood(20-34y)	237	35.91
Adulthood(35-59y)	362	54.85
Elderly(60-64y)	61	9.24
Average=41.38, SD=11.92		
Education level		
Primary school	286	43.33
High School	211	31.97
Diploma or equal	53	8.03
Bachelor degree	103	15.61
Higher than Bachelor degree	7	1.06
Occupation		
Agriculture	264	40
Trader	158	23.94
Laborer	103	15.61
Government official/enterprise/business	63	9.55
Out of work	39	5.91
Other	33	5
Religion		
Buddhism	410	62.12
Other	250	37.88
Marital status		
Single	108	16.36
Married/Partner	511	77.42
Widowed/Divorced/Separated	41	6.21
Family income		
Not enough and have debt	70	10.61
Not enough and no debt	37	5.61
Enough and no savings	365	55.3
Enough and have savings	188	28.48
Family history of cancer		
Yes	95	14.39
No	565	85.61
Family history of breast cancer		
Yes	17	2.58
No	643	97.42
Locality		
Rural	461	69.85
Urban	199	30.15

**Table 2:** Adjusted ORs and 95%CI of the Overall B-CAS (Overall) and B-CAS Subscale of Knowledge of Risk Factors of Breast Cancer (KnowRF) and Knowledge of Symptoms of Breast Cancer (KnowS). Categories in brackets represent the referent

Effects	Overall	KnowRF	KnowS
Age 10yrs	1.05 (0.90, 1.23)	1.01 (0.86,1.19)	1.22 (0.96,1.56)
Rural residence	0.43*** (0.29, 0.62)	0.40*** (0.27,0.59)	0.66 (0.37,1.17)
Education			
Primary school and lower	χ2=1.23	χ2=0.02	χ2=9.63
High School	1.03 (0.70.1.52)	1.09 (0.73,1.65)	1.71 (0.92,3.15)
Diploma or equal	1.17 (0.61, 2.22)	0.91 (0.48,1.73)	3.79* (1.22,11.82)
Bachelor degree	1.27 (0.70, 2.28)	1.92* (1.04,3.57)	2.46 (0.95,6.33)
Higher than Bachelor	0.67 (0.16, 2.80)	0.77 (0.14,4.11)	0.50 (0.08,3.26)
Occupation			
Agriculture	χ2=10.33	χ2=18.64**	χ2=12.26
Trader	1.11 (0.72, 1.72)	0.99 (0.62,1.57)	1.34 (0.63,2.86)
Laborer	0.68 (0.41, 1.14)	0.61 (0.36,1.03)	0.50 (0.24,1.04)
Government official	0.49* (0.24, 0.99)	0.25*** (0.11,0.53)	0.32* (0.11,0.94)
Unemployment	0.46* (0.23, 0.95)	0.41*** (0.19,0.87)	0.46 (0.16,1.30)
Other	0.46 (0.38, 1.65)	0.97 (0.44,2.14)	1.31 (0.36,4.79)
Other religion	0.94 (0.66, 1.32)	0.68 (0.47,0.98)	1.05 (0.61,1.79)
Marital status			
Single	χ2=6.94*	χ2=10.32**	χ2=5.11
Married	1.60* (1.03, 2.48)	1.52 (0.94,2.46)	1.99 (1.07,3.71)
Widowed/Divorced/Separated	0.90 (0.42, 1.91)	0.57 (0.26,1.24)	1.30 (0.46,3.67)
Family income			
enough and have savings	χ2=7.35	χ2=5.78	χ2=1.73
Enough and no savings	0.71 (0.49, 1.02)	0.64 (0.44,0.93)	0.91 (0.51,1.60)
Not enough and no debt	0.84 (0.41, 1.69)	0.71 (0.32,1.56)	1.06 (0.36,3.15)
Not enough and have debt	0.49* (0.29, 0.85)	0.63 (0.35,1.12)	0.59 (0.26,1.35)
Family history of cancer	0.89 (0.56, 1.41)	0.72 (0.45,1.15)	0.38* (0.16,0.95)
Family history of breast cancer	2.12 (0.77, 5.86)	1.42 (0.50,4.02)	1.09 (0.21,5.78)

χ2, Likelihood ratio test; ***P<0.001, **P<0.01,*P<0.05

**Table 3:** Adjusted ORs and 95%CI of B-CAS Subscale; Attitude to Breast Cancer Prevention (Attitude), Barriers of Breast Screening (Barriers), Health Behavior Related to Breast Cancer Awareness (Health Behavior). Categories in brackets represent the referent

Effects	Attitude	Barriers	Health Behavior
Age 10yrs	1.04 (0.88,1.22)	0.76* (0.64,0.91)	1.63*** (1.36,1.95)
Rural residence	0.60** (0.41,0.87)	0.56* (0.37,0.84)	4.73*** (3.08,7.27)
Education			
Primary school and lower	χ2=5.66	χ2=14.94**	χ2=43.72**
High School	1.42 (0.94,2.14)	0.44* (0.28,0.68)	3.80*** (2.42,5.96)
Diploma or equal	1.83 (0.94,3.56)	0.75 (0.37,1.52)	5.79*** (2.85,11.77)
Bachelor degree	1.83 (0.99,3.39)	0.51 (0.26,1.01)	2.67* (1.36,5.23)
Higher than Bachelor	1.17 (0.25,5.59)	1.20 (0.24,5.94)	5.02 (0.97,26.07)
Occupation			
Agriculture	χ2=10.37	χ2=6.83	χ2=12.96
Trader	1.07 (0.68,1.70)	0.99 (0.60,1.63)	0.54* (0.33,0.90)
Laborer	0.68 (0.40,1.16)	1.93* (1.09,3.41)	0.81 (0.46,1.43)
Government official	0.50 (0.24,1.07)	1.16 (0.51,2.62)	1.65 (0.75,3.65)
Unemployment	0.46* (0.22,0.98)	1.47 (0.66,3.27)	0.60 (0.28,1.32)
Other	0.53 (0.25,1.13)	1.13 (0.49,2.57)	0.56 (0.25,1.26)
Other religion	1.12 (0.77,1.61)	1.30 (0.88,1.92)	1.40 (0.95,2.06)
Marital status			
Single	χ2=0.81	χ2=0.55	χ2=3.46
Married	1.24 (0.77,2.00)	1.20 (0.72,2.00)	0.72 (0.43,1.21)
Widowed/Divorced/Separated	1.28 (0.56,2.90)	1.29 (0.55,3.02)	1.23 (0.53,2.86)
Family income			
enough and have savings	χ2=6.61	χ2=3.69	χ2=16.49***
Enough and no savings	0.69 (0.47,1.01)	0.86 (0.57,1.29)	0.72 (0.48,1.07)
Not enough and no debt	1.43 (0.68,3.00)	0.47 (0.21,1.08)	2.83** (1.30,6.15)
Not enough and have debt	0.74 (0.41,1.31)	1.05 (0.57,1.93)	1.36 (0.74,2.48)
Family history of cancer	1.09 (0.67,1.78)	1.43 (0.86,2.37)	1.18 (0.71,1.96)
Family history of breast cancer	2.40 (0.87,6.64)	1.10 (0.36,3.34)	0.54 (0.17,1.96)

χ2, Likelihood ratio test; ***P<0.001; **P<0.01; *P<0.05

## Discussion

Despite no substantial reduction in the incidence rate of breast cancer in developed countries, breast cancer mortality rates in these resource-sufficient countries has substantially decreased in the last few decades (Ferlay et al., 2010). However, this decrease in mortality rate has not flowed through to many developing countries suggesting both curative and preventive intervention, such as community level screening, are central to reducing breast cancer mortality. In many south-east Asian countries, no comprehensive population screening programs exist and later diagnosis of breast cancer is the result (Kim et al., 2015), and this is perhaps the most probable cause for the higher mortality rate observed in resource-limited settings. Instead, in resource-limited countries like Thailand, mammography tends to be limited to diagnosis (Virani et al., 2014) and breast self-examination remains one of the most common ways the disease is detected in the resource-limited setting. Breast cancer awareness, which aims to raise breast cancer knowledge in women, and stress the importance of breast self-examination and seeking clinical examination, is likely to lead to earlier detection, and in resource-limited settings, is likely to play an important role in decreasing breast cancer mortality. In this study, we use the validated B-CAS instrument to identify factors associated with breast cancer awareness in Thai women. 

We analyzed data from a community sample of Thai women and found that women living in rural areas had lower breast cancer awareness (the overall B-CAS scale) than women living in an urban setting. This result is consistent with some studies (Kanaga et al., 2011; Balouch et al., 2016), but Norlaili et al., (2013) in their study of Malay women found that rural women had a better level of breast cancer awareness compared to urban women. We also found married women had better breast cancer awareness relative to single women, even after adjusting for age. This aligns with the findings of Radi (2013) who also found married women to have higher breast cancer awareness. However, Parsa et al., (2008) in their study of a similar south-east Asian population (Malay women) found no association between marital status and breast cancer screening behaviors. This difference may be explained by subtle differences between Thai and Malay culture. For example, in Thai culture, it falls to daughters to look after their elder family members and it may be that younger women strongly motivate their mothers to seek regular medical checkups. 

Ultimately, breast cancer awareness behavior is one of the most important domains considered in the present study. It is this domain that ultimately leads to higher incidence of breast self-examination and seeking clinical examination. One of the most striking results in the present study was that rural women, despite poorer breast cancer knowledge and attitude, were considerably more likely to exhibit better breast cancer health behavior (diet, physical activity, seeking clinical examination and breast screening) than urban women. While other studies have found that rurality is positively associated with breast cancer awareness behavior in Australian women (Leung et al., 2014) and Malaysian women (Norlaili et al., 2013), no other study has observed such a strong effect size; we found that Thai rural women have almost 5 times the odds of better breast cancer health behavior relative to their urban counterparts. However, it should also be noted that there is inconsistency in the literature about rurality being positively associated with better breast cancer health behavior. Kanaga et al., (2011) reported that rural women had poorer breast cancer practices compared to urban women in Malaysia. Similar to that observed in other populations (such as Iran and the Unitied Arab Emirates), we found that age was also positively associated with better breast cancer health behavior (Elobaid et al., 2014; Tilaki et al., 2015). However, Parsa’s et al., (2008) study of breast cancer awareness among Malaysian women could not demonstrate age to be associated with breast cancer health behavior. We also found that women with higher education had better breast cancer health behavior. This has been demonstrated in several other studies of various populations (Gurdal et al., 2012; Norlaili et al., 2013; Donnelly et al., 2014; Tilaki et al., 2015) and confirms that general education plays an important role of health literacy, at least for breast cancer. 

This study did have some limitations. Women included in our study were limited to two southern provinces of Thailand, and whether our sample is representative of all Thai women is unknown. Furthermore, we employed a cross-sectional study design and while it makes sense that the relatively non-modifiable demographic factors we considered would necessarily precede the breast cancer awareness outcomes we studied, nevertheless such study designs are limited regarding statements of causality. The present study also had a major strength. We used a well-developed breast cancer awareness instrument that has been validated in Thai women (Rakkapao et al., 2016, 2017) and other south-east Asian populations (Solikah et al., 2017). 

Interestingly, our analysis found the two most important factors, those significantly associated with most breast cancer awareness domains, to be rurality and age. Higher age has been demonstrated across many studies as being associated with many breast cancer awareness domains. However, our results regarding the associations of rurality and the various B-CAS domains was surprising. In particular, despite women living in rural areas having both poorer knowledge of breast cancer symptoms and poorer attitudes to breast cancer screening, these same women had a substantially higher likelihood of better breast cancer behavior. It is not clear why this might be, but it suggests that interventions with an emphasis of disease knowledge may not be the best approach to improving breast cancer health behaviors such as breast self-examination and seeking clinical examination. It was true that we observed that rural women perceived fewer barriers to breast cancer screening than their urban counterparts, and this may be due to lifestyle differences between rural and urban Thai women. 

Despite the methods used for early detection differing in the two settings, the role of breast cancer awareness in both resource-sufficient and resource-limited countries is very important in decreasing the breast cancer mortality rate. Specifically, breast cancer awareness is likely to increase health-seeking behavior. Consequently, we are left with the challenge of how to raise breast cancer awareness to ensure the best possible outcomes. Somewhat surprisingly, in the present study we found that while rural women had lower knowledge of breast cancer risk factor, they had substantially better breast cancer awareness behavior and a lower level of perceived barriers to breast cancer health care. This suggest interventions wholly aimed at enhancing disease knowledge are unlikely to be effective in increasing breast self-examination among Thai women. Instead emphasis should be placed on access to breast cancer health services, and the importance of breast self-examination as a trigger for seeking clinical examination.
